# High rate of dyspareunia and probable vulvodynia in Ehlers–Danlos syndromes and hypermobility spectrum disorders: An online survey

**DOI:** 10.1002/ajmg.c.31939

**Published:** 2021-11-07

**Authors:** Jennifer E. Glayzer, Barbara L. McFarlin, Marco Castori, Marie L. Suarez, Monya C. Meinel, William H. Kobak, Alana D. Steffen, Judith M. Schlaeger

**Affiliations:** ^1^ Department of Human Development Nursing Science College of Nursing, University of Illinois Chicago Chicago Illinois USA; ^2^ Division of Medical Genetics Fondazione IRCCS‐Casa Sollievo Della Sofferenza San Giovanni Rotondo Italy; ^3^ Department of Obstetrics and Gynecology College of Medicine, University of Illinois Chicago Chicago Illinois USA; ^4^ Department of Population Health Science College of Nursing, University of Illinois Chicago Chicago Illinois USA

**Keywords:** dyspareunia, Ehlers–Danlos syndromes, pelvic pain, social media, vulvodynia

## Abstract

Vulvodynia is debilitating vulvar pain accompanied by dyspareunia (pain with sexual intercourse). Ehlers–Danlos syndromes (EDS) and hypermobility spectrum disorders (HSD) may represent a predisposing factor for vulvodynia given a high rate of dyspareunia in these conditions. We conducted an online survey of women with EDS or HSD to assess rates of dyspareunia and estimate rates of vulvodynia, report rates of comorbid conditions common to EDS or HSD and vulvodynia, and examine rates of conditions contributing to dyspareunia in women with EDS or HSD. Women with EDS or HSD (*N* = 1,146) recruited via social media were 38.2 ± 11.5 years old, primarily White (94.4%), and resided in the United States (78.5%). 63.7% of participants reported dyspareunia and 50% screened positive for vulvodynia. The rate of comorbid conditions common to EDS or HSD and vulvodynia were: irritable bowel syndrome, 6.5%; fibromyalgia, 40.0%; temporomandibular joint dysfunction, 56.4%; migraine, 6.7%; interstitial cystitis, 1.7%; and mast cell activation syndrome, 10.2%. Participants reporting dyspareunia also reported ovarian cysts, fibroids, or abdominal or pelvic scars, 47.5%; endometriosis, 26.5%; and genital lacerations, 19.3%. Women with EDS or HSD may have a higher rate of vulvodynia (50.0%) than women in the U.S. population at large (8%) and should be assessed for dyspareunia and vulvodynia.

## 1 INTRODUCTION

Pain is nearly universal in hypermobility spectrum disorders (HDS) (Castori & Hakim, 2017; Demes, McNair, & Taylor, 2020), is a diagnostic criterion of hypermobile Ehlers–Danlos syndrome (hEDS) (Malfait et al., 2017), and is common in adults with rarer types of EDS (Schubart, Schaefer, Hakim, Francomano, & Bascom, 2019; Voermans, Knoop, Bleijenberg, & van Engelen, 2010). Co‐morbidities of EDS are equally common in HSD and hEDS (Copetti et al., 2019). Given the evolving use of the terms hEDS (Tinkle et al., 2017), HSD (Castori et al., 2017), and joint hypermobility syndrome (Grahame, Bird, & Child, 2000) an “old” term incorporated in HSD (Grahame et al., 2000), in this paper, we used the acronym “hEDS/HSD” to identify the community of phenotypes belonging to these three, partially overlapping groups. In hEDS and presumably HSD, as an individual ages recurrent injuries accumulate resulting in chronic pain, with hypothesized central and/or peripheral nervous system sensitization (Castori, 2016; Sacheti et al., 1997). Pain is estimated to affect 90% (Voermans et al., 2010) of individuals with hEDS/HSD and has such a profound effect that even with pain management 87% (Voermans et al., 2010) report difficulties performing activities of daily living (Castori, 2016). Phenotypic dimensions of pain in EDS are heterogeneous and still incompletely understood.

Women with EDS were reported to have an alarming 77% rate of dyspareunia (pain with sexual intercourse) (Castori et al., 2012; Hugon‐Rodin, Lebegue, Becourt, Hamonet, & Gompel, 2016; Hurst et al., 2014) compared to 20% (Latthe, Latthe, Say, Gülmezoglu, & Khan, 2006; Seehusen, Baird, & Bode, 2014) of women in the general global population. Vulvodynia, debilitating chronic vulvar pain accompanied by dyspareunia (Bornstein et al., 2016), affects up to 8% (Reed, Harlow, Sen, Legocki, et al., 2012), of women in the United States and has no identifiable underlying pathology. Desperate for pain relief women with vulvodynia may go as far as having a vulvectomy to relieve their pain, knowing it is possible that pain may still continue (Andrews, 2011; Goldstein et al., 2016; Tommola, Unkila‐Kallio, & Paavonen, 2010). Several EDS studies have categorized dyspareunia and vulvodynia as the same condition (Castori et al., 2012, 2017; Castori, Morlino, et al., 2013); however, more specifically, dyspareunia is a symptom of vulvodynia. No studies have examined vulvodynia in women with EDS or HSD (Castori et al., 2012; Castori, Morlino, et al., 2013; Chopra et al., 2017; Gilliam, Hoffman, & Yeh, 2020; Hugon‐Rodin et al., 2016; Hurst et al., 2014; McIntosh, Mallett, Frahm, Richardson, & Evans, 1995).

Due to the rare nature of EDS and the lack of healthcare specialists familiar with EDS and HSD, patients have used Facebook™ (Meta Platform Inc, Menlo Park, CA) to form support groups where tips and resources are shared. In these groups, we observed women complaining of dyspareunia. Pain from dyspareunia can decimate a person's life, rendering them incapable of having sexual intercourse, shattering an intimate relationship (Schlaeger, Pauls, et al., 2019). A review of the literature found five studies that reported the rate of dyspareunia in EDS and HSD but no studies examined the conditions that may contribute to dyspareunia in EDS and HSD (Castori et al., 2012; Chopra et al., 2017; Hugon‐Rodin et al., 2016; Hurst et al., 2014; McIntosh et al., 1995). In the general population, vulvodynia; endometriosis; pelvic inflammatory disease; infection and neurological conditions affecting the genitals; ovarian cysts, fibroids, and scarring; previous injury to the genitals; laceration of the genitals; vaginal dryness; atrophic vaginitis; chemotherapy and radiation; and cancer have been found to be associated with dyspareunia (Alimi, Iwanaga, Oskouian, Loukas, & Tubbs, 2018; American College of Obstetricians and Gynecologists & Committee on Gynecologic Practice, 2016; Seehusen et al., 2014; Sorensen, Bautista, Lamvu, & Feranec, 2018). Separately, it is known that EDS, HSD, and vulvodynia are both associated with comorbid conditions such as irritable bowel syndrome (Maeland, Assmus, & Berglund, 2011; Reed, Harlow, Sen, Legocki, et al., 2012; Vieira‐Baptista, Lima‐Silva, Cavaco‐Gomes, & Beires, 2014), fibromyalgia (Reed, Harlow, Sen, Edwards, et al., 2012; Vieira‐Baptista et al., 2014), temporomandibular joint dysfunction (Murray, Yashar, Uhlmann, Clauw, & Petty, 2013; Vieira‐Baptista et al., 2014), interstitial cystitis (Castori, Celletti, & Camerota, 2013; Reed, Harlow, Sen, Edwards, et al., 2012; Vieira‐Baptista et al., 2014), mast cell disorders (McDonald & Rapkin, 2012; Regauer, Eberz, & Beham‐Schmid, 2015; Seneviratne, Maitland, & Afrin, 2017), and migraine (Hakim & Grahame, 2004; Puledda et al., 2015; Vieira‐Baptista et al., 2014). The intersections of these comorbid conditions have never been reported in the EDS, HSD, or vulvodynia literature. The primary aim of this study was to determine the rate of dyspareunia and vulvodynia in women with EDS or HSD. The secondary aim was to report the rate of comorbid conditions that are common in both EDS, HSD, and vulvodynia. The third aim was to examine the rate of conditions known to contribute to dyspareunia in women with EDS or HSD.

## 2 METHODS AND MATERIALS

The study was a cross‐sectional online self‐reported survey that was completed from June to July of 2019. This study was approved by the University of Illinois Chicago Institutional Review Board.

**FIGURE 1:**
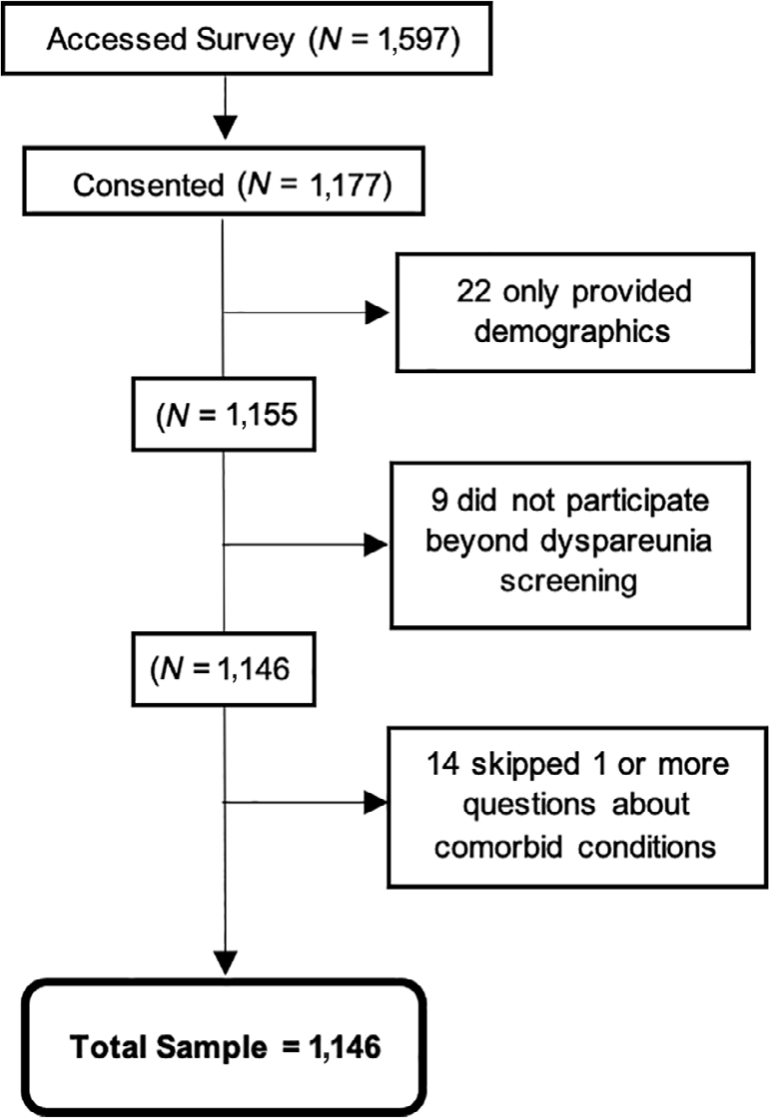
Participation flow chart

A convenience sample of participants was recruited through social media and met the inclusion criteria of: (1) a self‐reported diagnosis of EDS or HSD previously confirmed by a healthcare provider; (2) assigned to the female sex at birth and not had genital gender reassignment surgery; (3) 18 years of age or older; and (4) able to read English. Figure 1 illustrates participation in the study. A total sample of 1,146 participants were used for all calculations. Fourteen participants skipped occasional questions regarding conditions associated with dyspareunia but were kept in the final count due to the small percentage of missing data.

The survey was conducted using Qualtrics (Qualtrics®, Provo, UT) and was accessed via a link posted in EDS Facebook support groups and on Twitter (Twitter Inc, San Francisco, CA). Of the 171 EDS support groups approached on Facebook, 55 gave permission to post our survey link once. The survey was posted with the title *Ehlers–Danlos Syndrome and Women's Health Issues* to prevent participants from ascertaining that the survey was focused on dyspareunia and vulvodynia which could skew participation. Furthermore, the survey description encouraged women with and without women's health issues to participate to accurately reflect the presence of women's health issues in women with EDS or HSD. The same study description and link was also posted on Twitter with the hashtag #EDS. Individuals that were interested in this survey selected the link via either Facebook or Twitter and were brought to Qualtrics where electronic consent was obtained. Qualtrics prevented participants from completing the survey more than once by limiting participation to one per Internet Protocol address. Qualtrics deidentified data available to researchers by removing the IP addresses.

**FIGURE 2:**
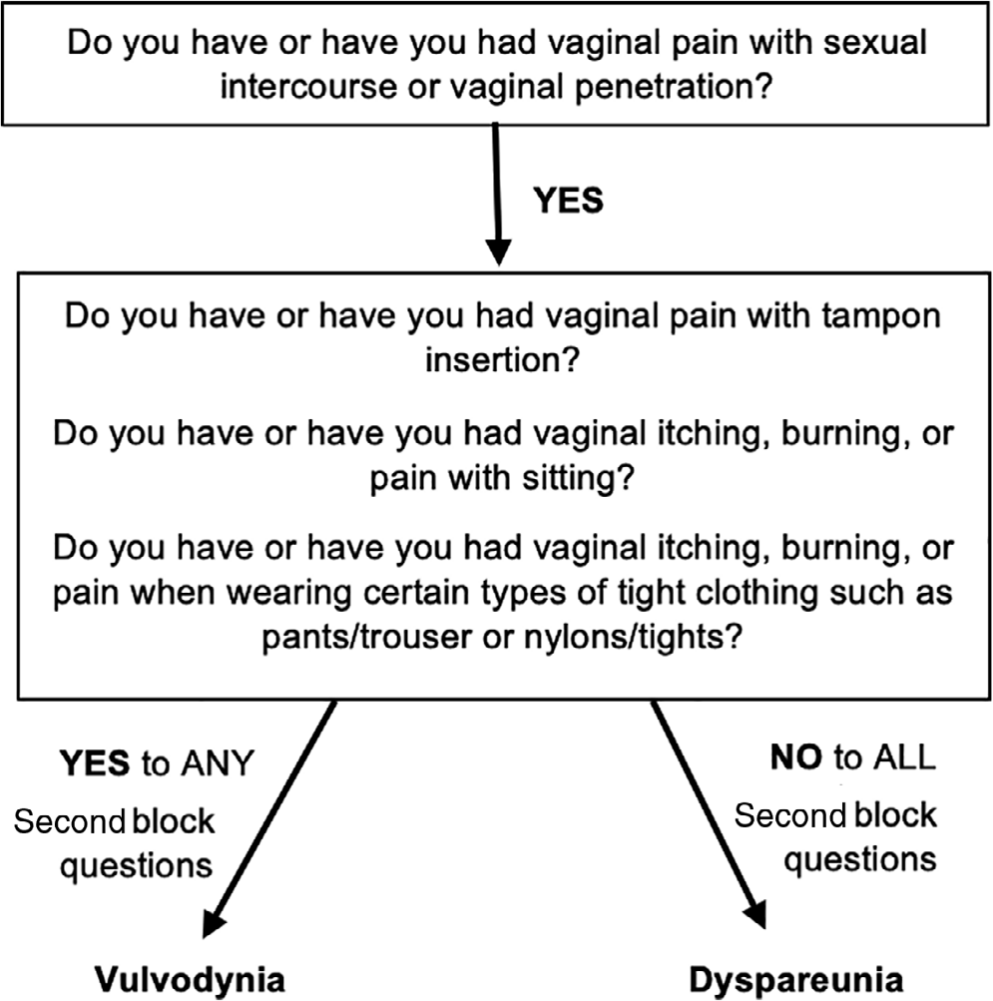
Vulvodynia screening criteria adapted from Reed, Haefner, Harlow, Gorenflo, and Sen (2006) and Harlow et al. (2009)

This survey was developed based on a previously validated online survey used to evaluate dyspareunia and vulvodynia (Schlaeger, Patil, et al., 2019). For this study, dyspareunia was defined as painful vaginal sexual intercourse; vulvodynia was defined as chronic vulvar pain accompanied by dyspareunia. The survey consisted of multiple choice and open‐ended questions. Multiple choice questions asked about participant's demographics, type of EDS, comorbid conditions associated with EDS, HSD, and vulvodynia, conditions associated with dyspareunia, and vulvodynia symptoms. Questions regarding conditions associated with dyspareunia had response options of yes/no/I'm not sure. All conditions included are shown in Figure 4 (Alimi et al., 2018; American College of Obstetricians and Gynecologists & Committee on Gynecologic Practice, 2016; Seehusen et al., 2014; Sorensen et al., 2018). Open‐ended questions were used to follow up on “I'm not sure” responses providing space for the participant to elaborate on their specific situation. Comorbid conditions shared by EDS or HSD and vulvodynia were selected from a list of choices with an additional write‐in option to include conditions that were not listed. The presence of vulvodynia was determined in the sample using two methods: (1) Vulvodynia Screening Criteria (Figure 2) and (2) participant's self‐report of a vulvodynia diagnosis. Both methods were used to ascertain the rate of vulvodynia in this often‐underdiagnosed chronic pain syndrome. We developed the Vulvodynia Screening Criteria based on four vulvodynia symptoms including dyspareunia, found to be valid and reliable at diagnosing vulvodynia in two surveys (one online and one that used paper questionnaires) in lieu of a pelvic exam (Harlow et al., 2009; Reed et al., 2006). The Vulvodynia Screening Criteria developed for this study was used in a previous vulvodynia study (Schlaeger, Patil, et al., 2019) and was adapted from surveys by Reed et al. (2006) and Harlow et al. (2009). Reed et al.'s (2006) survey had a Cohen's *ĸ* of 0.78 (95% confidence interval [CI] 0.64–0.92) with accuracy of 96.4% for vulvodynia diagnoses, with the survey compared to an in‐office visit. Reed et al.'s (2006) survey also had 94.1% accuracy in determining subjects without vulvodynia. Our survey ranged from 29 to 56 questions using branch logic (depending on reported symptomatology) and took an average of 10–20 min to complete, with only deidentified data collected.

Data were exported from Qualtrics into Microsoft Excel for cleaning and coding prior to analysis using Stata Software for Statistics 15 (StataCorp LLC, College Station, TX). Missing data were minimal (<3%) in the analysis sample, suggesting minimal bias to our results. Listwise deletion was used in cases with missing responses. Descriptive statistics were used to summarize and describe data results. Correlation tables were used to assess for multicollinearity. Logistic regression was used to describe the effect of demographics and EDS type on a participant's odds of having dyspareunia and vulvodynia. Logistic regression was also used to describe the effect of comorbid conditions in common between EDS (or HSD) and vulvodynia on a participant's odds of having vulvodynia (Table 2). The *α* level was set at <0.05. “I'm not sure” follow‐up questions were assessed by a vulvodynia expert (J.S.) and were left as “I'm not sure” or changed to “Yes” or “No” based on the response.

## 3 RESULTS

**FIGURE 3:**
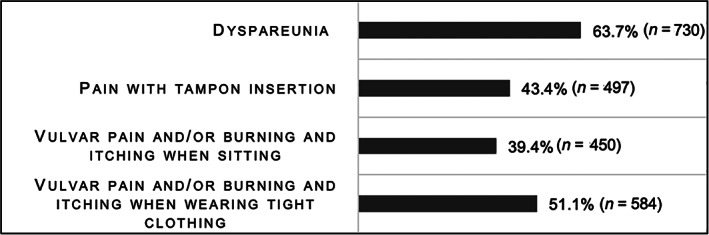
Vulvodynia symptoms in women with Ehlers–Danlos syndromes and hypermobility spectrum disorders. Each question had a slightly different sample size due to missing data. Sample sizes range from 1,142 to 1,155

**FIGURE 4:**
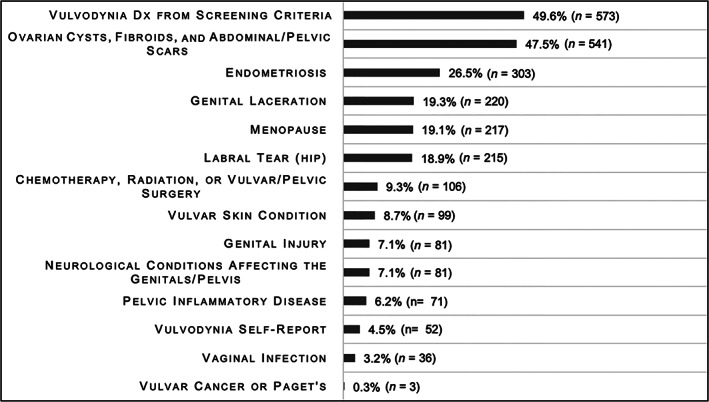
Prevalence of conditions associated with dyspareunia in women with Ehlers–Danlos syndromes and hypermobility spectrum disorders. Each question had a slightly different sample size due to missing data. Sample sizes range from 1,137 to 1,146

The survey was accessed by 1,597 potential participants with 1,177 women consenting to participate. Of the 1,177 participants 1,146 completed the survey with <3% (*n* = 14) skipping occasional questions regarding vulvodynia symptoms and conditions known to be associated with dyspareunia. Participants had a mean age of 38.2 years (±11.5 years *SD*), were predominantly White, from the United States, and most had a diagnosis of hEDS/HSD. Of the 14 EDS types, 8 were represented; with hEDS/HSD, classical EDS (cEDS), and vascular EDS (vEDS) being the most common types (Table 1). Eighty‐eight percent of participants resided in either the United States or England, and 12% in 1 of 27 other countries. Characteristics of the sample are summarized in Table 1. The rate of dyspareunia was determined by the response to the question “Do you have, or have you had pain with sexual intercourse?”. Next, 63.7% (*n* = 730) of all participants reported pain with intercourse and 3.6% (*n* = 42) of all participants reported they were virgins or not sexually active and were unable to determine if they had dyspareunia. Of the 42 (3.6%) participants who reported they were virgins or not sexually active, 15 reported pain with tampon insertion. The rate of vulvodynia was determined using the Vulvodynia Screening Criteria (Figure 2) with 573 (50%) participants screening positive. The rate of each vulvodynia symptom is shown in Figure 3. Figure 4 shows the rate of each condition associated with dyspareunia. Vulvodynia, ovarian cysts, fibroids, abdominal and pelvic scars, and endometriosis were the most common conditions associated with dyspareunia in our sample.

**TABLE 1:** Characteristics of sample (*N* = 1,146)

Demographics	*N*	%	Country	*N*	%	EDS type	*N*	%
*Age*			United States	900	78.5	Hypermobile/hypermobility spectrum disorders	1046	91.3
Mean	1,146	38.2 ± 11.5	England	111	9.7	Classic	50	4.4
Range	1,146	18–77	Canada	46	4.0	Not sure which type	21	1.8
Menopausal	217	18.9%	Australia	23	2.0	Vascular	10	0.9
			Scotland	8	0.7	Classical‐like	9	0.8
*Gender*			Belgium	6	0.5	Kyphoscoliotic	6	0.5
Female	1,144	99.8	Ireland	6	0.5	Cardiac‐valvular	2	0.2
Transgender	2	0.2	Norway	5	0.4	Arthrochalasia	1	0.1
			South Africa	5	0.4	Myopathic	1	0.1
Race^a^			Wales	5	0.4	Brittle cornea	0	0
White	1,063	92.8	The Netherlands	4	0.3	Dermatosparaxis	0	0
Black or African American	12	1.0	New Zealand	4	0.3	Musculocontractural	0	0
American Indian or Alaskan Native	17	1.5	Sweden	3	0.3	Periodontal	0	0
Asian	11	1.0	Denmark	2	0.2	Spondylodysplastic	0	0
Native Hawaiian or Pacific Islander	3	0.3	France	2	0.2			
Other	47	4.1	Germany	2	0.2			
			Italy	2	0.2			
*Ethnicity* (*N* = 1,138)			Jordan	2	0.2			
Hispanic/Latino	41	3.6	Countries with one participant	10	1.0			
Not Hispanic/Latino	1,046	91.9						
Unknown or Not Reported	51	4.5						

Abbreviation: EDS, Ehlers–Danlos syndromes.

^a^ Participants selected all races they identified with.

**FIGURE 5:**
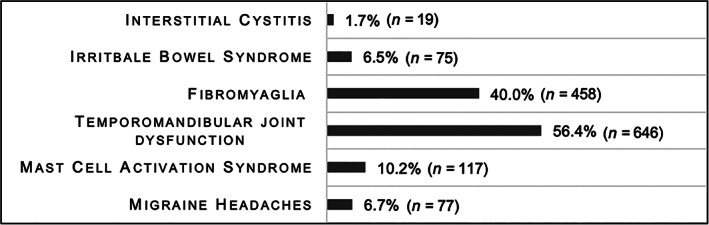
Frequency of comorbid conditions associated with vulvodynia and Ehlers–Danlos Syndromes or hypermobility spectrum disorders (*N* = 1,146)

Irritable bowel syndrome (Maeland et al., 2011;Reed, Harlow, Sen, Edwards, et al., 2012; Vieira‐Baptista et al., 2014), fibromyalgia (Reed, Harlow, Sen, Edwards, et al., 2012; Vieira‐Baptista et al., 2014), temporomandibular joint dysfunction (Murray et al., 2013; Vieira‐Baptista et al., 2014), interstitial cystitis (Castori, Celletti, et al., 2013; Reed, Harlow, Sen, Edwards, et al., 2012; Seneviratne et al., 2017), mast cell activation syndrome (McDonald & Rapkin, 2012; Regauer et al., 2015; Seneviratne et al., 2017), and migraine (Hakim & Grahame, 2004; Puledda et al., 2015; Vieira‐Baptista et al., 2014) are known comorbidities of EDS and vulvodynia. Figure 5 shows the number of each comorbid condition in the total sample with fibromyalgia having the highest rate at 40% (*n* = 458).

**FIGURE 6:**
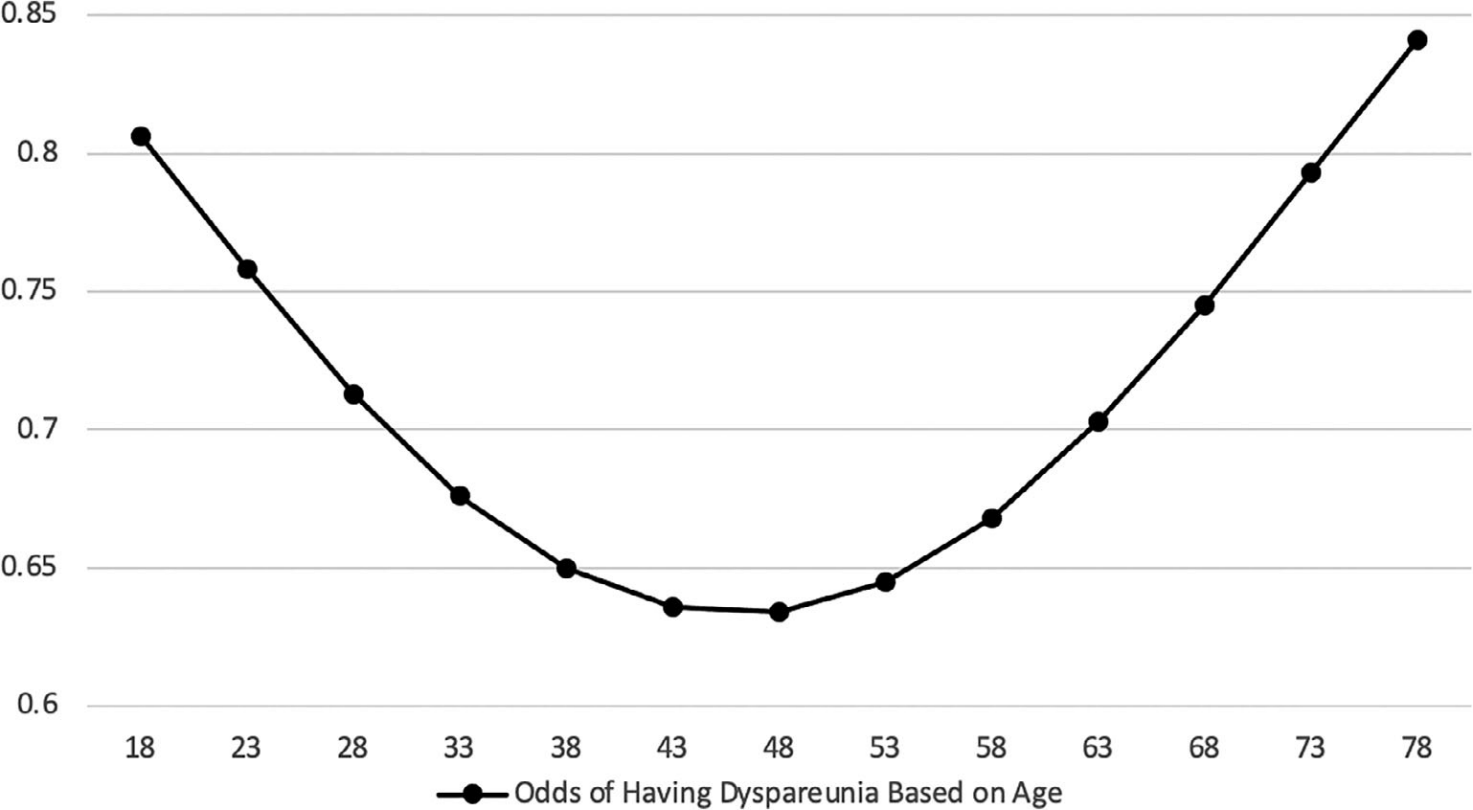
Logistic odds of having dyspareunia based on age (*N* = 1,118). Sample size of 1,118 due listwise deletion of missing observations

Participants' demographics were significantly associated with the odds of whether or not they had dyspareunia or screened positive for vulvodynia (Table 2). The odds of having dyspareunia varied significantly based on age and whether or not a participant was White (*p* = .006). Age had a nonlinear relationship with the odds of women having dyspareunia. Younger women and older women had higher odds of having dyspareunia than middle age women; with women in their mid‐40s having the lowest odds (Figure 6). White participants had decreased odds of having dyspareunia compared to participants who were not White. Similarly, the odds of screening positive for vulvodynia varied significantly based on demographics. Age had a linear relationship with the odds of screening positive for vulvodynia, with each 1‐year increase in age the odds decreased. Participants in the United States had decreased odds of screening positive for vulvodynia compared to participants who resided outside the United States. The odds of having dyspareunia or screening positive for vulvodynia did not vary based on whether or not a participant was hispanic or latino, or if the participant had hEDS/HSD compared to other types of EDS. The effect of specific races other than White, countries outside of the United States, and EDS types not including hEDS/HSD on the odds of reporting dyspareunia or screening positive for vulvodynia were unable to be evaluated due to small group size.

**TABLE 2:** Logistic odds of having dyspareunia and vulvodynia based on demographics (*N* = 1,146)

Variable	Adjusted OR	95% CI	*p*
*Dyspareunia* (*p* = .006)*			
Age	0.902	0.841–0.968	.004*
U.S. resident	0.836	0.464–1.506	.550
White	0.708	0.511–0.980	.037*
hEDS	0.855	0.542–1347	.499
Hispanic or Latino	1.232	0.747–2.033	.414
*Vulvodynia* (*p* = .001)*			
Age	0.981	0.977–0.998	.018*
U.S. resident	0.668	0.498–0.895	.007*
White	0.848	0.498–1.444	.533
hEDS	0.768	0.506–1.167	.216
Hispanic or Latino	1.491	0.947–2.349	.085

Abbreviations: CI, confidence interval; hEDS, hypermobilie Ehlers–Danlos syndrome; OR, odds ratio.

* Significant findings at *p* < .05 level.

The odds of participants with EDS or HSD screening positive for vulvodynia varied significantly based on which comorbid conditions shared EDS or HSD and vulvodynia (*p* < .001) (Table 3) they had when adjusting for age and residing in the United States. Participants that had fibromyalgia, interstitial cystitis, temporomandibular joint dysfunction, migraine headaches, or interstitial cystitis had increased odds of screening positive for vulvodynia (Table 3). However, the sample of participants with interstitial cystitis was small (*n* = 19). Irritable bowel syndrome and mast cell activation syndrome did not correlate with screening positive for vulvodynia.

**TABLE 3:** Logistic odds of having vulvodynia based on the presence of comorbid conditions common in Ehlers–Danlos syndromes (and hypermobility spectrum disorders) and vulvodynia (*p* = .000)

Variable	*N*	Adjusted OR	95% CI	*p*
Age	1,146	0.984	0.974–0.995	.003*
U.S. resident	1,146	0.639	0.476–0.858	.002*
Temporomandibular joint dysfunction	651	1.286	1.008–1.639	.043*
Fibromyalgia	463	1.885	1.457–2.416	.000*
Migraine headaches	78	1.548	0.939–2.554	.087
Mast cell activation syndrome	118	0.928	0.622–1.384	.714
Interstitial cystitis	19	5.652	1.607–19.883	.007*
Irritable bowel syndrome	75	0.632	0.381–1.048	.075

* Significant findings at *p* < .05 level.

## 4 DISCUSSION

The two new findings in this study are: (1**)** women with EDS or HSD may have a higher rate of vulvodynia (50%) than women in the U.S. population at large (8%); and (2) EDS or HSD and vulvodynia have comorbid conditions in common. Dyspareunia is a hallmark of vulvodynia. Our sample had a high rate of dyspareunia (63.7%) that was consistent with three previous studies on EDS that reported it to be as high as 77% (Castori et al., 2012; Hugon‐Rodin et al., 2016; Hurst et al., 2014). However, this study also examined the rate of other conditions associated with dyspareunia in women with EDS (Castori et al., 2012; Hugon‐Rodin et al., 2016; Hurst et al., 2014; Tinkle et al., 2017). Our finding that 50% of women with EDS or HSD may also have vulvodynia, over six times the rate of the general population, is important because this group has a high chronic pain burden (Schlaeger, Patil, et al., 2019; Schlaeger, Pauls, et al., 2019; Tinkle et al., 2017; Voermans et al., 2010). Women with EDS have fragile lax tissues (*Ehlers–Danlos Syndrome*: *National Library of Science* [*US*], 2019; Malfait et al., 2017; Tinkle et al., 2017) and report spontaneous vaginal lacerations due to the poor integrity of their collagen which may promote scarring and dyspareunia. Lax ligaments in EDS and HSD can cause pelvic instability (Aydeniz et al., 2010; Hunt, Clohisy, & Prather, 2007; Tinkle et al., 2017) which can result in hypertonic pelvic floor muscles in an attempt to stabilize the pelvis (Hartmann & Sarton, 2014; Morin et al., 2017; Prather, Dugan, Fitzgerald, & Hunt, 2009). Hypertonic pelvic floor muscles have been found to contribute to vulvodynia (Bornstein et al., 2016), which may explain the high rate of vulvodynia in women with EDS and HSD. Our data suggest that these women suffer chronic pain that interferes with their intimate relationships.

The four questions used to screen for vulvodynia in lieu of a pelvic exam: (1) Do you have or have you had pain with sexual intercourse or vaginal penetration? (2) Do you have or have you had vaginal pain with tampon insertion? (3) Do you have or have you had vaginal itching and burning or pain with sitting? And (4) Do you have or have you had vaginal itching and burning or pain when wearing certain types of tight clothing such as pants/trousers or nylons/tights? (Vulvodynia Screening Criteria) (Harlow et al., 2009; Reed et al., 2006) were instrumental in enabling us to perform this survey in a large sample of women from around the world. Identifying that dyspareunia occurs and vulvodynia may occur at a higher rate in EDS and HSD highlights the importance of ensuring adequate gynecologic screening for dyspareunia.

Demographics may have an impact on the odds of having dyspareunia and vulvodynia. The U‐shaped curve (Figure 6) relationship between age and the odds of having dyspareunia may be explained by the increased odds of vulvodynia in younger women (<48 years old) and the increased odds of atrophic vaginitis in postmenopausal women (>48 years old). We found a linear relationship between age and the odds of screening positive for vulvodynia, where a woman's odds of screening positive decreased as a woman aged. Younger women may be at higher risk for screening positive for vulvodynia due to the use of combined estrogen‐progestin oral contraceptives, which may be associated with a higher rate of vulvodynia (Bornstein et al., 2016). Decreased odds for vulvodynia in women who reside in the United States may be explained by culturally specific issues that may influence the manifestation of vulvodynia which requires further examination. Whether or not women had hEDS/HSD or another type of EDS did not impact the odds of dyspareunia or vulvodynia. Our findings suggest that women with EDS and HSD should be screened for dyspareunia and vulvodynia, and that dyspareunia and vulvodynia impact this group universally.

It is well known that women suffering from chronic pain conditions may have a higher prevalence of shared comorbid conditions such as fibromyalgia, temporomandibular joint disorder, irritable bowel syndrome, interstitial cystitis, migraine, and vulvodynia (Backonja & Argoff, 2005; Biasi, Di Sabatino, Ghizzani, & Galeazzi, 2014; Bornstein et al., 2016; Bouhassira et al., 2005; Puledda et al., 2015; Reed, Harlow, Sen, Edwards, et al., 2012). It is hypothesized this is due to sensitization of the central and or peripheral nervous system (Backonja & Argoff, 2005; Camerota, Celletti, Castori, Grammatico, & Padua, 2011; Castori, 2016). However, our study identified that these same comorbid conditions are indeed shared by women with EDS or HSD and vulvodynia. Comorbid conditions have been reported in EDS and HSD (Hakim & Grahame, 2004; Maeland et al., 2011; Murray et al., 2013; Puledda et al., 2015; Seneviratne et al., 2017) and women with vulvodynia (McDonald & Rapkin, 2012; Regauer et al., 2015; Vieira‐Baptista et al., 2014) separately, but never together. Women with fibromyalgia, temporomandibular joint dysfunction, migraine, or interstitial cystitis had higher odds of having dyspareunia and vulvodynia than those without the respective comorbid conditions. This finding suggests that both EDS or HSD and vulvodynia may have a neuropathic component which affects treatment options (Bornstein et al., 2016; Castori, Celletti, et al., 2013; Castori, Morlino, et al., 2013; Schlaeger, Patil, et al., 2019). Clinical presentation is typically complex, with multiple comorbidities, rendering it unlikely that an individual or cohort would respond to one single treatment option.

Our study's large sample size is noteworthy and would have not been possible in a prospective design. A limitation of this study was that all diagnoses were self‐reported or diagnosed through a reliable and validated screening tool and were not verified by a healthcare provider (Harlow et al., 2009; Reed et al., 2006). Using self‐report and a screening tool to examine the rate of conditions could increase the rate of false positives. However, the burden of performing pelvic exams would have made the large sample size difficult, if not impossible to obtain. In addition, our findings are roughly in line with the “real world” experience in clinics and daily living of affected individuals, which documents a wide array of chronic pain manifestations in people with EDS and HSD.

Conducting EDS research is difficult as most types are classified as a rare disease (National Insititutes of Health, 2017; Steinmann, Royce, & Superti‐Furga, 2002) which hampers case finding. Using social media enabled us to examine gynecological conditions in women with EDS or HSD in a large population from around the globe, thus suggesting the utility and benefit of using an online survey format. Our sample was a convenience sample of only women who had access to providers that diagnosed EDS or HSD, used social media, and could read English. Participants in this study were predominantly from the United States, White, had internet access, and used social media; and the study was conducted in English. The Vulvodynia Screening Criteria enabled us to identify women who may have vulvodynia in a large sample who also have a rare disease.

## 5 CONCLUSION

The findings of our study suggest important information for clinicians caring for women with EDS or HSD. EDS, HSD, and vulvodynia have shared comorbid conditions that include fibromyalgia, interstitial cystitis, and temporomandibular joint dysfunction. The complex nature of EDS, HSD, and vulvodynia make their treatment a clinical challenge. The first step toward treatment is recognizing that women with EDS or HSD may have vulvodynia and dyspareunia. Recognizing the high rate of vulvodynia in this population may provide insights into risk factors for vulvodynia as well as the development of new treatments.

## CONFLICT OF INTEREST

The authors have no conflicts of interest to disclose.

## Data Availability

The data that support the findings of this study are available from the corresponding author upon reasonable request.
